# A Novel Disorder of Osteoporosis, Osteonecrosis, and Metaphyseal Fracture

**DOI:** 10.1002/jbm4.10365

**Published:** 2020-04-28

**Authors:** Hans‐Georg Zmierczak, Guy Taylor, Tim Cundy

**Affiliations:** ^1^ Department of Endocrinology Ghent University Hospital Ghent Belgium; ^2^ Department of Rheumatology Whanganui Hospital Whanganui New Zealand; ^3^ Department of Medicine, Faculty of Medical and Health Sciences University of Auckland Auckland New Zealand

**Keywords:** BISPHOSPHONATES, METAPHYSEAL FRACTURES, OSTEONECROSIS, OSTEOPOROSIS

## Abstract

We describe two unrelated women who in their fifth decade developed a severe disorder characterized by large joint osteonecrosis and multiple minimal trauma fractures in both the axial and appendicular skeleton, including unusual metaphyseal fractures of the proximal tibia. Bone density testing showed borderline osteoporosis of the spine and osteopenia of the femur. Therapy with bisphosphonates and teriparatide failed to prevent further fractures. To our knowledge, this disorder has not been described previously. Investigations to date, including a genetic screen, have not revealed its cause. © 2020 The Authors. *JBMR Plus* published by Wiley Periodicals, Inc. on behalf of American Society for Bone and Mineral Research.

## Introduction

Osteonecrosis (avascular necrosis) of long bones is most commonly associated with trauma or prolonged high‐dose steroid use, particularly in the context of lupus, human immunodeficiency virus infection, bone marrow transplantation, or solid organ transplantation. Other causes include sickle cell disease, Morquio syndrome, and Gaucher disease.^1^ Idiopathic osteonecrosis is rare, but has been associated with thrombophilic disorders and alcohol dependence. We describe two unrelated women who in their fifth decade developed a severe bone disorder characterized by both osteonecrosis and multiple osteoporotic fractures. To our knowledge, this appears to be a novel disorder.

## Patients and Methods

### Case histories

#### Subject A

**Figure 1:**
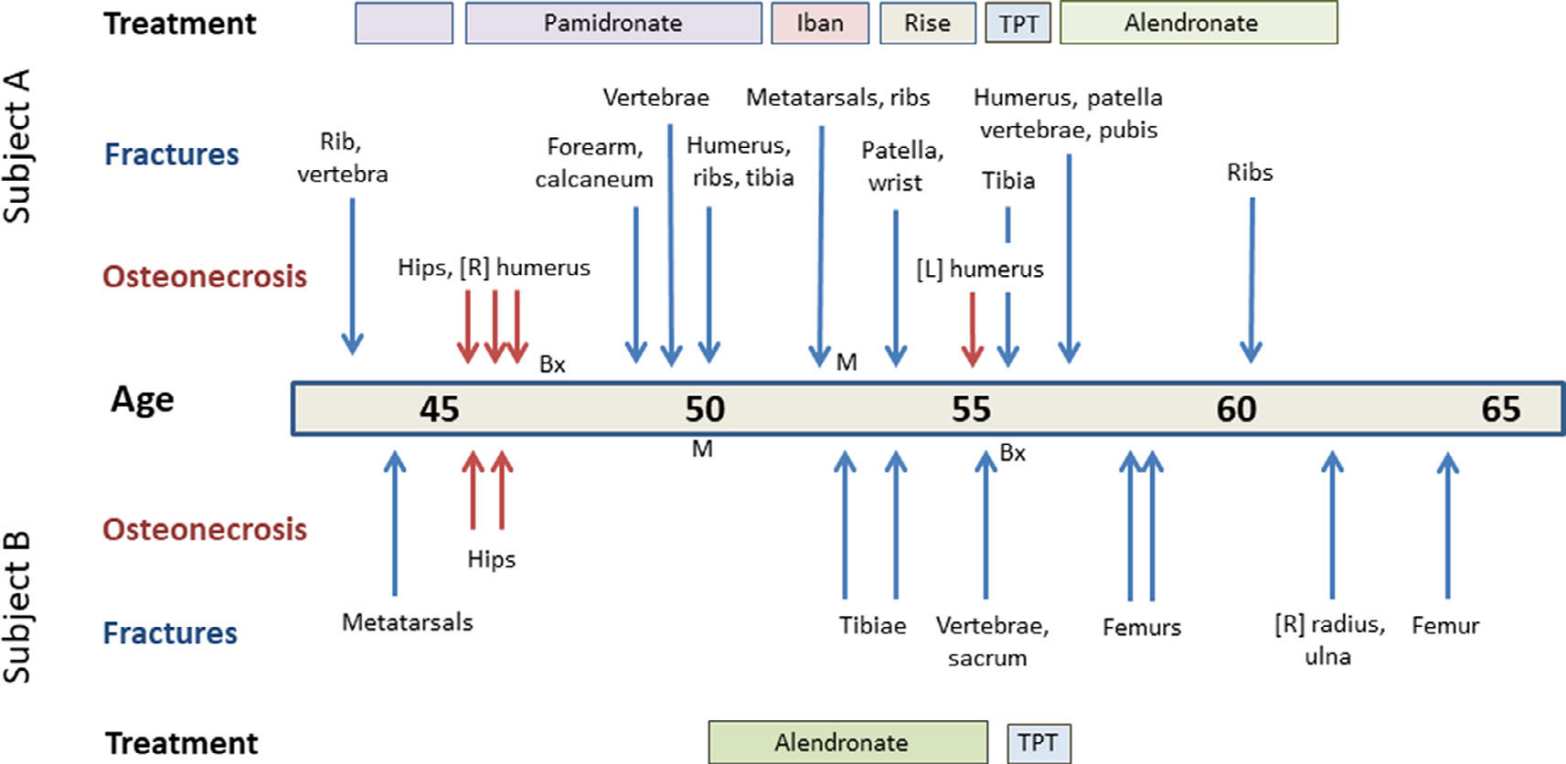
Timeline to indicate age at occurrence of fractures and episodes of osteonecrosis and treatment periods in subjects A (upper panel) and B (lower panel). M = time of menopause; Bx = time of bone biopsy; Iban = ibandronate treatment; Rise = risedronate treatment; TPT = teriparatide treatment.

At the age of 42 subject A, who was of European descent, presented with a rib fracture and shortly after, a vertebral fracture. Osteoporosis was diagnosed and i.v. pamidronate treatment was started. In the next 3 years she developed, in rapid succession, osteonecrosis of both hips and her right humeral head. Over the following 10 years, despite continued treatment with various bisphosphonates, she sustained multiple long bone, metatarsal, pubic, rib, and vertebral fractures after minimal trauma (see Fig. 1 for chronology). The tibial fractures were unusual, occurring through the metaphyseal region. At the age of 55 she developed osteonecrosis of the left humeral head and had joint replacement surgery.

She had sustained one fracture as a 9‐year‐old, but otherwise had no significant past medical history. She had one uneventful pregnancy. She had never smoked. There was no history of an eating disorder. Her BMI at presentation was 19.6 kg/m^2^. She had been of normal stature (1.62 m), but by age 55 had lost 15 cm of height through multiple vertebral fractures. She reached menopause at the age of 53. There was no history of bone disease in her parents or siblings. She had no dysmorphic features or signs of osteogenesis imperfecta or cortisol excess. She had no history of thromboembolic disease. At age 43 she was recognized to have mild dyslipidemia; she was taking a low dose of atorvastatin to bring her cholesterol level to normal. Her alcohol intake was moderate. At age 63 she is ambulant without assistance, but restricted in walking longer distances and activities that involve raising the arms above shoulder level.

#### Subject B

At the age of 44 subject B, who was also of European descent, developed metatarsal fractures. The following year she experienced sudden onset of pain in the right groin. Radiographs showed disintegration of the femoral head, most likely caused by avascular necrosis, and she had a hip joint replacement. Just a few months later she experienced severe pain on the left side with similar findings on radiography, and again required hip joint replacement surgery (see Fig. 1 for chronology). She took alendronate from age 50 and remained reasonably well until age 53, when she developed sudden pain below the left knee. Radiography showed a metaphyseal fracture, causing the leg to bow outwards. Surgical treatment with an upper tibial osteotomy was undertaken. Shortly afterward, she developed sudden onset of similar pain just below the right knee. After 6 weeks the pain settled, but there was outward bowing of the right leg. Just over a year later the pain worsened and she was unable to weight‐bear. She subsequently had an osteotomy and repair through what was presumed to be a fracture that had healed with deformity. Spine radiographs at age 55 showed vertebral fractures. She later broke both distal femurs and suffered a periprosthetic femoral fracture on the left.

She reported no childhood fractures, and there was no family history of fracture or bone disease. She had an inguinal hernia repair at the age of 8 and an appendicectomy at the age of 9. She had three uneventful pregnancies. She smoked until the age of 45. There was no history of an eating disorder. Her BMI at presentation was 22.0 kg/m^2^. Dyslipidemia (total cholesterol = 9.7 mmol/L) was recognized at age 46, but she declined statin therapy until age 52. At the age of 50, she had a hysterectomy, oophorectomy, and temporary defunctioning colostomy for sepsis related to a tubo‐ovarian abscess. She had no history of thromboembolic disease. She had been of normal stature (1.61 m) prior to the onset of vertebral fractures, but between the ages of 48 and 64 she lost 5 cm of height. She had no dysmorphic features or signs of osteogenesis imperfecta or cortisol excess. Her alcohol intake was moderate. At the age of 65 she remains ambulant and manages all her activities of daily living, but slowly and carefully.

## Results

### Laboratory investigations

Basic hematological and biochemical investigations (including renal function, thyroid function, plasma calcium, phosphate, calcidiol, PTH, alkaline phosphatase (ALP), and liver enzymes (including gamma‐glutamyl transpeptidase) were normal in both subjects. Bone resorption markers prior to bisphosphonate treatment were not measured. Biochemical testing for Cushing syndrome was negative. Gaucher disease was excluded by the finding of normal leukocyte β‐glucocerebrosidase activity (Table 1). Systemic mastocytosis, myeloma, celiac disease, and antiphospholipid syndrome were excluded by appropriate testing. Antinuclear antibodies were negative. Plasma homocysteine (measured only in subject B) was normal.

**Table 1:** Biochemical Findings

	Subject A	Normal values/units	Subject B	Normal values/units
Alkaline phosphatase	41^a^	30 to 120 U/L	81	30 to 120 U/L
β‐glucocerebrosidase activity	4.41 μmol/L/h	1 to 5 μmol/L/h	945 pmol/min/mg protein	600 to 3200 pmol/min/mg protein
Cushing syndrome screening	9 a.m. plasma cortisol 210 nmol/L	110 to 550 nmol/L	9 a.m. plasma cortisol 348 nmol/L With normal diurnal variation	110 to 550 nmol/L
Homocysteine	**‐**	‐	7.5 μmol/L	5 to 15 μmol/L

a Result obtained on bisphosphonate treatment.

### Radiology

**Figure 2:**
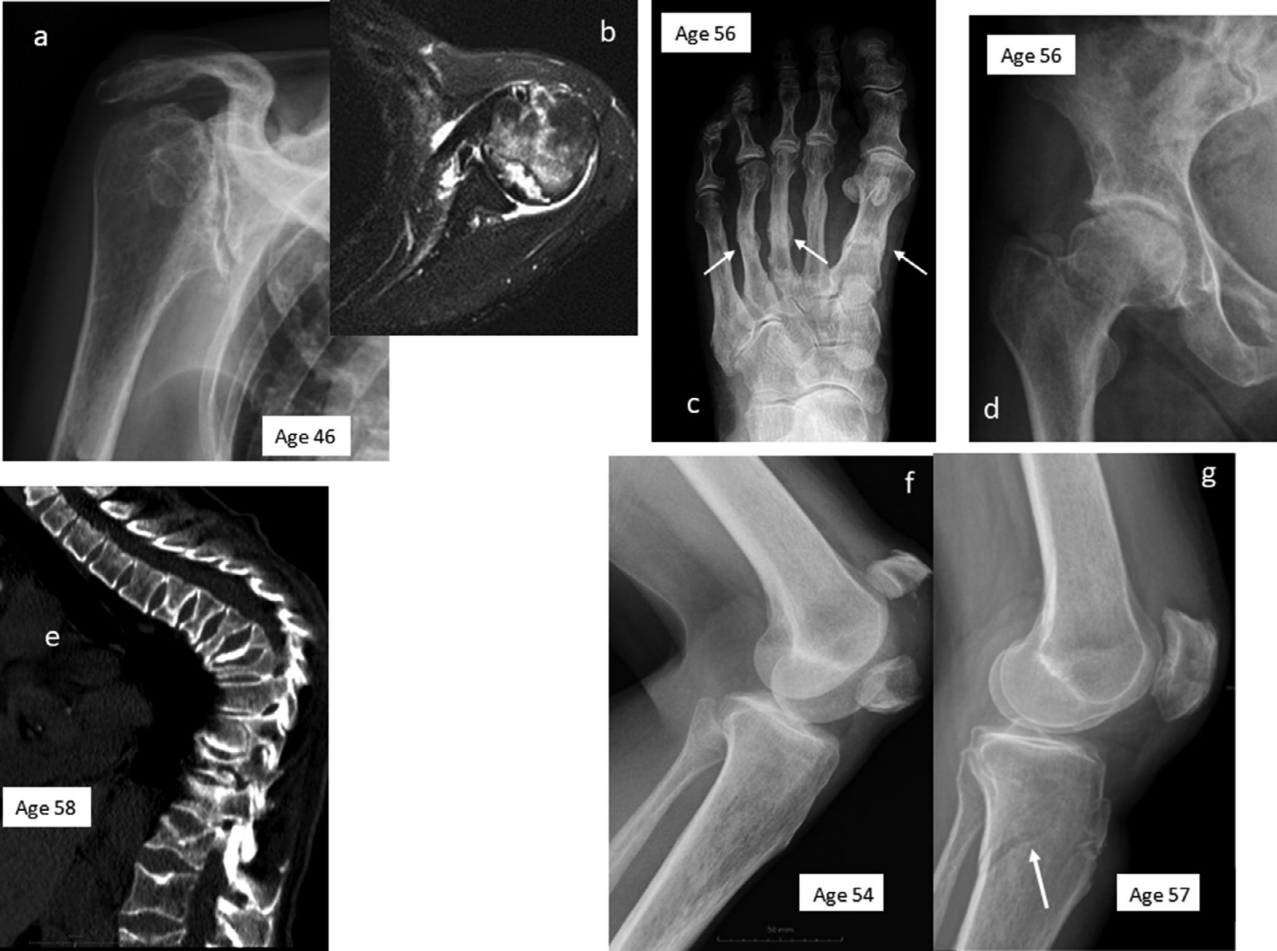
Selected radiographs from subject A. (*A*,*B*) Radiograph and MRI illustrating osteonecrosis of the shoulder, (*C*) metatarsal fractures (arrows), (*D*) osteonecrosis of the femoral head, (*E*) multiple vertebral fractures (MRI), (*F*) patellar fracture, also showing spotty osteoporosis of the upper tibia, and (*G*) submetaphyseal tibial fracture (arrow). The subject's age at the time of each radiograph is indicated.

**Figure 3:**
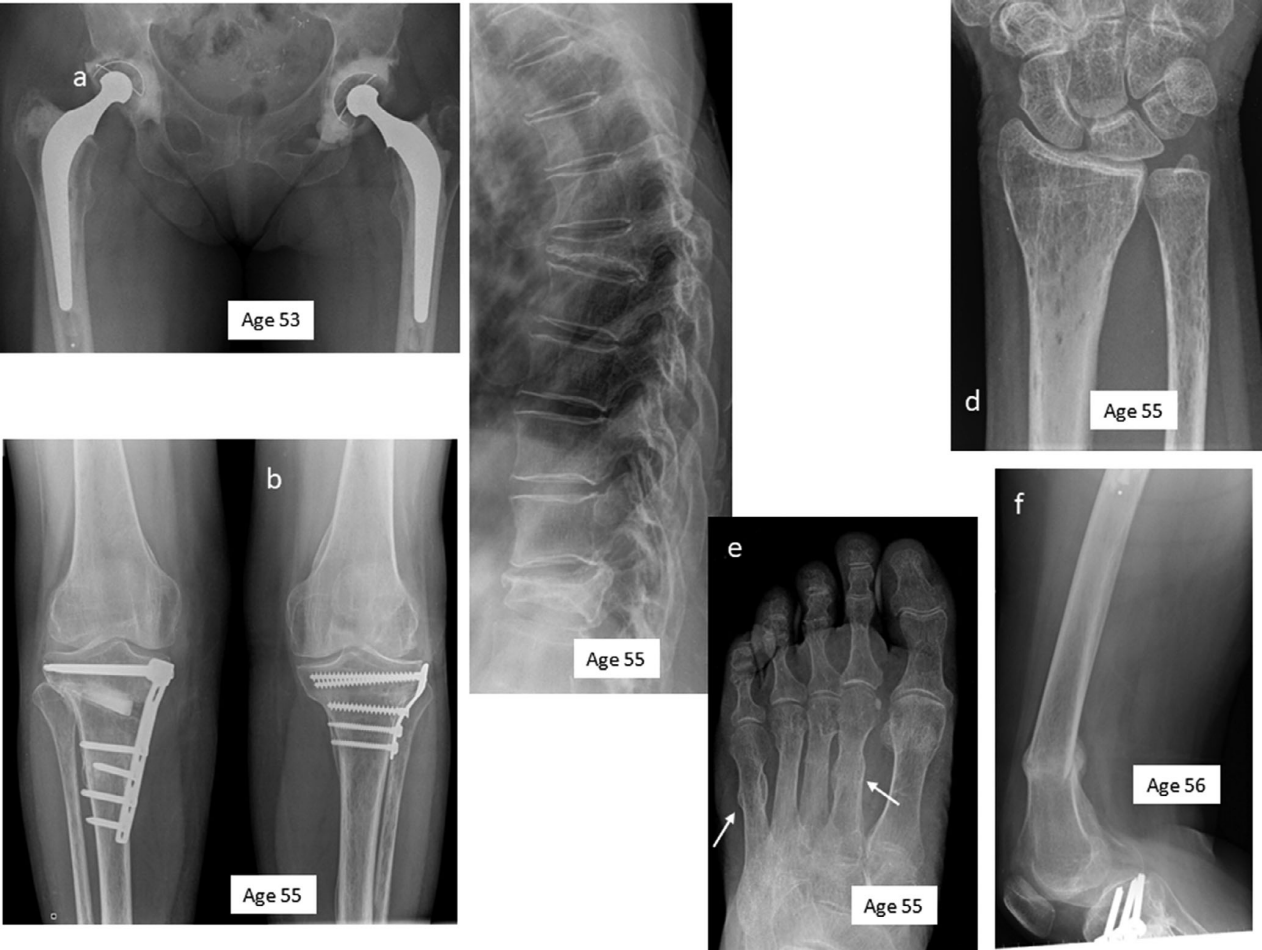
Selected radiographs from subject B. (*A*) Bilateral hip joint replacements after osteonecrosis of the hips, (*B*) bilateral osteotomy repairs for submetaphyseal tibial fractures, (*C*) vertebral fractures, (*D*) spotty osteoporosis of the distal radius and ulna, (*E*) healing metatarsal fractures (arrows), and (*F*) distal femoral fracture. The subject's age at the time of each radiograph is indicated.

The long bone cortices of subject A were thin, but there were no Looser zones. Almost all the thoracic vertebras showed grade 2 or grade 3 fractures at the time of the most recent imaging. Her humeral heads, femoral heads, and tibial plateaus were deformed, and there were signs of past metatarsal, pubic, clavicular, and rib fractures (Figures 2A‐G). On review at age 55, subject B had several vertebral compression fractures. The long bones showed thin cortices and spotty osteoporotic changes. There were healing fractures of the inferior ischiopubic rami and lateral bowing of the tibia and fibula, particularly distally. The bones in the feet had thin cortices, with evidence of previous metatarsal fractures (Figures 3A‐F). Skull radiographs were normal in both subjects. A bone scintiscan (subject B) showed increased isotope uptake in all the areas affected by recent lesions, but no other abnormalities.

### Bone densitometry

Both women had bone density measurements before starting bisphosphonate treatment. The results were similar with borderline osteoporosis at the lumbar spine and osteopenia at the femoral neck. The respective *T*‐scores were A (age 42): −2.5 and − 1.3; B (age 45): −2.4 and −1.6.

### Bone biopsy

The femoral head from subject B excised at age 45 showed disorganization of the chondro‐osseous junction with fibrous granulation tissue, necrotic bone, and new bone formation. The features were consistent with avascular necrosis.

Transiliac bone biopsies from both subjects were taken and undecalcified sections prepared. Compared with published normal values,^2^ both showed thin cortices, increased cortical porosity, and low trabecular bone volume, but no signs of osteomalacia. In both cases, bone turnover appeared low, but the biopsies had been obtained after 5 years of bisphosphonate treatment. There was no histological evidence of systemic mastocytosis.

Formal quantitation was undertaken only in subject B's biopsy. The trabecular bone volume was reduced and trabecular thickness low. The cortical bone showed increased cortical porosity. Trabecular osteoid was reduced (Table 2). Few osteoblasts were observed. A short length of tetracycline double‐labeling was seen in cortical bone, but none was seen in trabecular bone.

**Table 2:** Quantitative Bone Histology From Subject B at Age 55

Parameter		Result	Normal values^2^
Trabecular bone volume	BV/TV	11.0%	22.5 ± 3.5
Cortical thickness	Ct.Th	567 μm	909 ± 98
Cortical porosity	Ct.PO	17.2%	6.3 ± 0.6
Trabecular osteoid volume	OV/BV	0.6%	1.9 ± 0.4
Trabecular osteoid thickness	O.Th	2.05 μm	9.5 ± 0.6
Trabecular eroded surface	ES/BS	5.4%	5.1 ± 0.6

### Genetic screening

Screening for mutations in 29 genes associated with bone fragility was undertaken using a next‐generation sequencing platform. Genomic DNA from leukocytes of subject A was enriched with SureSelectXT Low Input Human All Exon V7 (Agilent Technologies, Santa Clara, CA, USA) followed by sequencing on a HiSeq 3000 platform (Illumina, San Diego, CA, USA). The genes examined were: *ACAN, ALPL, B3GALT6, BMP1, COL1A1, COL1A2, CREB3L1, CRTAP, FAM46A, FKBP10, IFITM5, LEPRE1, LRP5, LRP6, MBTPS2, NBAS, P4HB, PLOD2, PLS3, PPIB, SEC24D, SERPINF1, SERPINH1, SP7, SPARC, TAPT1, WNT1, TMEM38B,* and *LIFR*. No pathogenic variants were detected, though the technique used is less sensitive for detecting deletions and duplications >15 bp, repeat expansions, and copy number variants.

### Response to treatment

Both patients had intensive bisphosphonate treatment (see Fig. 1), but continued to fracture. Both were subsequently treated with an 18‐month course of teriparatide injections, but again, fractures continued to occur. Subject A was also treated with alfacalcidol while taking bisphosphonates, but this was discontinued during teriparatide treatment because of hypercalcemia. Bisphosphonate treatment was given after teriparatide to subject A, but subject B declined further treatment. With teriparatide treatment, the bone formation markers ALP and procollagen‐1 N‐propeptide increased as expected in subject A (data for subject B incomplete).

As indicated above and in Fig. 1, fractures continued despite bisphosphonate and teriparatide treatment. In subject A sequential measures of spinal bone density were difficult to interpret because of progressive vertebral deformities, but the femoral neck bone density *T*‐score fell between the ages of 48 and 65 from −1.3 to −2.6. In subject B, sequential measures of bone density were not possible because of hip replacement surgery and progressive vertebral deformities, but the forearm bone density *T*‐score fell between the ages of 53 and 59 from −1.2 to −4.1.

## Discussion

To our knowledge this disorder of osteoporosis with osteonecrosis has not been described previously. The two women, who were unrelated and had no occupational exposure to toxins, had strikingly similar clinical presentations with fractures and osteonecrosis of the hip joints that began in their mid‐40s, before menopause. In both, fractures of the long bones and vertebra and further osteonecrosis episodes continued despite therapy with bisphosphonates, and later, teriparatide. Bone density testing close to the onset of the illness showed borderline osteoporosis at the lumbar spine and osteopenia at the femoral neck, but despite intensive therapy with bisphosphonates bone loss occurred during follow‐up. The femoral fractures sustained by subject B did not meet the criteria for bisphosphonate‐related atypical femur fractures, as suggested by the ASBMR taskforce.^3^ Both women sustained fractures of the proximal tibial metaphyses. Metaphyseal fractures of this type (41‐A2/A3 in the AO classification) are rare, comprising only 3% of tibial fractures.^4^ The majority occurs after trauma in men under the age of 40, so apparently spontaneous fractures in women are exceptional, and in our cases it is possible that the osteonecrotic process underlay these fractures.

The bone biopsies, taken after bisphosphonate treatment, showed osteoporosis with no sign of osteomalacia, and gave no pathological clues. The conditions most commonly linked to both osteoporosis and osteonecrosis are sickle cell disease, Cushing syndrome, and Gaucher disease. Neither subject had clinical evidence of sickle cell disease, cortisol excess, nor was taking exogenous steroids. Though usually diagnosed earlier in life, Gaucher disease can present in the fifth decade, but neither woman had anemia, splenomegaly, or Erlenmeyer flask deformities of the femurs, and both had negative biochemical tests for this disorder. Of note, both women had high‐serum cholesterol (severe in subject B). There is anecdotal data that hyperlipidemia might be a risk factor for the development of osteonecrosis in children with acute lymphoblastic leukemia^5^ and that statin therapy might lower the risk of osteonecrosis in people treated with corticosteroids.^6^ However, neither osteoporosis nor osteonecrosis are associated with familial hyperlipidemia, suggesting that the latter alone is unlikely to explain the disorder we describe.

The lack of response to bisphosphonate therapy and continued fractures with osteonecrosis suggests perhaps osteoblast or osteocyte failure, although fractures did seem to heal. Neither subject had a family history of any similar disorder, and whether this is an acquired condition or genetic we do not know. However, it would be unusual for a genetic bone disorder to present for the first time this late in life, and in one subject screening of 29 genes known to be associated with bone fragility was negative. We would be interested to hear from other clinicians who have seen similar cases.

## Disclosures

The authors declare no conflicts of interest.
